# Revision Distal Biceps Tendon Repair Using Original Intramedullary Buttons

**DOI:** 10.1016/j.eats.2022.11.006

**Published:** 2023-01-18

**Authors:** Bryan Adams, Bobby Yow, Christopher Daniels, Emily Morgan

**Affiliations:** a John A. Feagin, Jr. Sports Medicine Fellowship, Keller Army Community Hospital, West Point, New York; b Madigan Army Medical Center, Tacoma, Washington, U.S.A.

## Abstract

There are several techniques used for tendon fixation in distal biceps tendon repair. Intramedullary unicortical button fixation has the advantage of high biomechanical strength, minimal proximal radial bone removal, and low risk of injury to the posterior interosseous nerve. One disadvantage in revision surgery is retained implants in the medullary canal. This article describes a novel technique for revision distal biceps repair initially fixed with intramedullary unicortical buttons, using the original implants.

The distal biceps tendon has a vascular watershed near its insertion on the radial tuberosity.^1^ It receives its blood supply from the brachial artery proximally and the recurrent radial artery distally, making it prone to rupture.^1^ Distal biceps tendon injuries are most commonly seen in males in their fourth decade of life.^2^ The mechanism of injury is typically an eccentric load when the elbow is forced from a flexed to an extended position.^2^ Diagnosis is made on physical examination with occasional advanced imaging to determine whether the injury is a partial versus complete rupture.^2^ Many of these injuries are treated with surgery because the population they occur in are typically active, and 30% to 50% loss of supination strength is not well tolerated in this group.^2^

The surgical technique used in distal biceps repair varies widely. The primary benefit of the single anterior incision is reduced incidence of heterotopic ossification compared to the dual incision technique.^3^ The primary benefits of the dual incision technique are reduced risk to the posterior interosseous nerve and better access to the anatomic footprint on the radial tuberosity.^4^ The most common complication is injury to the lateral antebrachial cutaneous nerve, seen in 8% to 9% of all cases and seen slightly more often in the single anterior incision technique.^4^ Fixation techniques include bone tunnels, interference screw, cortical button, suture anchor, and combination of cortical button with interference screw.

Recently, fixation of the distal biceps with 2 intramedullary cortical buttons has been described.^5^^,^^6^ The primary benefit of this technique is avoiding drilling of the far cortex of the proximal radius and thus minimizing injury to the posterior interosseous nerve. The dual button technique allows the surgeon to recreate the footprint of the long and short head insertions on the radial tuberosity.^5^^,^^6^ Little has been written regarding revision techniques for distal biceps injuries treated with intramedullary cortical buttons. We present a revision technique that minimizes further bone loss and uses previously placed intramedullary buttons.

## Patient Evaluation

Acute distal biceps tendon ruptures typically occur when the tendon experiences excessive eccentric forces. Patients may describe a pop with immediate onset of pain about the elbow when attempting to lift heavy objects or catch a falling object. Physical examination may demonstrate ecchymosis around the antecubital fossa, proximal retraction of the biceps muscle (Popeye sign), inability to hook the lateral border of the distal biceps tendon (hook test), and weakness with elbow flexion or supination. In patients who have had a previous distal biceps tendon repair, it is important to specifically examine the lateral antebrachial cutaneous nerve and posterior interosseous nerve. These are the nerves most commonly injured in distal biceps repair, and any deficits noted before surgery may prompt the surgeon to explore the nerve at the time of revision surgery. The surgeon must also know the original approach used (dual vs single incision), the orientation of the original incision (longitudinal vs transverse), and type of fixation used (suspensory button, suture anchor, bone tunnels, interference screw, etc.) to properly plan revision distal biceps repair.

## Imaging

Plain radiographs allow the surgeon to assess the integrity and location of implants used at the index surgery. Radiographs will also show the amount of bone available in the proximal radius for fixation at the time of revision. Advanced imaging with magnetic resonance imaging will allow the surgeon to assess the integrity of the distal biceps tendon and degree of tendon retraction. This is critical in helping the surgeon determine whether an additional allograft is needed to augment the repair at the time of revision.

## Indications

In general, nonsurgical management is reserved for low-demand elderly patients and those who are not medically fit for surgery. In a healthy, active patient, 30% to 50% loss of supination strength is not well tolerated, and surgical management is typically recommended.

## Technique

**Fig 1:**
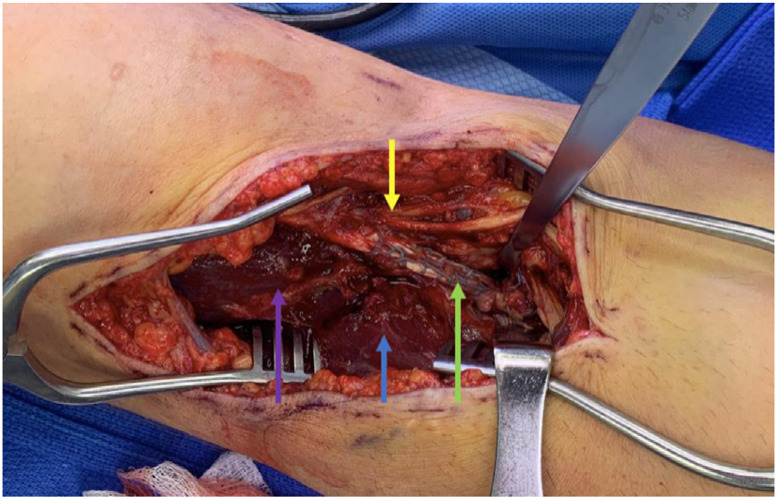
This is an intraoperative photo at the time of revision surgery. The patient is positioned supine on the gurney with a hand table, the head is to the left, and hand is to the right of the image, the superior part of the picture is medial and inferior part is lateral. Iatrogenic medial transposition and entrapment of the lateral antebrachial cutaneous nerve is demonstrated (yellow arrow) medial to distal biceps tendon (green arrow). Brachialis and brachioradialis are noted by purple and blue arrows, respectively.

This describes our technique in a patient who requires revision of a distal biceps repair that was originally repaired with 2 unicortical intramedullary buttons (Video 1). The patient is positioned supine on the hospital gurney with a hand table attachment. The previous anterior incision is used, and dissection is carried down between the brachioradialis and pronator teres to the repair site (Fig 1).

The initial suture knot is carefully taken down using a dental pick, and the whipstitched suture is removed from the distal biceps tendon, taking care to preserve the free tails of the suture. Once the suture is removed from the tendon, the unicortical drill holes are cleared with a small curette.

**Fig 2:**
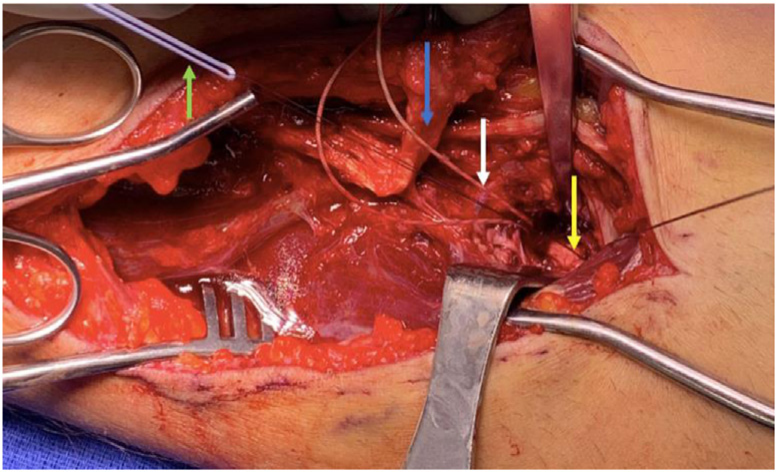
The patient’s head is to the left of the image, hand is to the right; superior in the image is medial, and the inferior part of the image is lateral. The previously placed repair suture has been atraumatically removed from distal biceps tendon (blue arrow) using a dental pick and forceps. A nylon suture lasso from a labral suture passing device (yellow arrow) has been passed through the intramedullary button, and a new repair suture (green arrow) will be shuttled back through button. A new suture (white arrow) has already been shuttled through the other button using the 2-0 Vicryl shuttling technique.

A 2-0 absorbable braided suture on an SH needle (Vicryl, SH; Ethicon, Bridgewater, NJ) is passed through the old no. 2 braided suture twice to reduce the chance of the Vicryl sliding out. The other end of the original no.2 braided suture is pulled to shuttle the Vicryl through the holes in the intramedullary button. Once shuttled through, the same needle on the Vicryl suture is passed through a new no. 2 braided suture 2 times. The needle on the Vicryl suture is then removed, and the tail of the Vicryl suture is pulled to shuttle the new No. 2 braided suture through the intramedullary button. Thus, we are able to maintain the original intramedullary button with new suture to revise the distal biceps repair. This is repeated for the second button. Note that a new No. 2 suture can also be shuttled through the old button utilizing a looped nylon from an arthroscopic labral repair suture passer. This is described here to demonstrate another viable option for shuttling of new suture (Fig 2).

**Fig 3:**
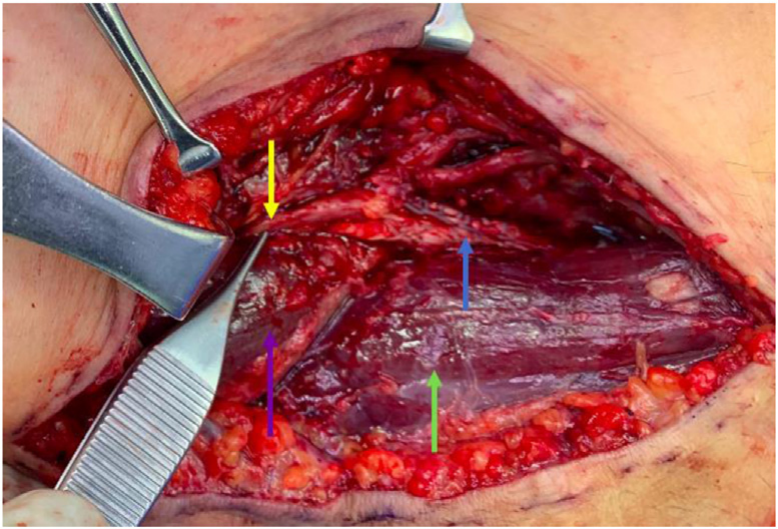
The patient’s head is to the left of the image, and hand is toward the right. This demonstrates that the lateral antebrachial cutaneous nerve (yellow arrow) is now lateral to the distal biceps tendon (blue arrow) after revision distal biceps repair. Brachialis (purple arrow) and brachioradialis (green arrow) are noted.

A running locking stitch is placed through the tendon with one limb of the suture. It is then shuttled down to the proximal radius by pulling on the free post and is tied in place with the elbow flexed with an arthroscopic knot pusher and the revision is complete using the 2 original intramedullary buttons (Fig 3). After surgery the patient is placed in a long arm splint for 1 week, the splint is then removed, and the patient begins early range of motion under therapist supervision.

## Discussion

The unicortical intramedullary button technique used for fixation of the distal biceps in this patient was described by Camp et al.^5^ in 2016. It has been further validated in several biomechanical studies demonstrating equivalent and in some cases, superior biomechanical strength than suture anchor and extramedullary button fixation.^6^^,^^7^ The greatest advantage of this revision technique is a safe and effective method to use the original intramedullary buttons. This technique could also be performed in other areas of the body where cortical button fixation is utilized such as a revision proximal biceps tenodesis or revision pectoralis major tendon repair. Another advantage of this technique is that there is no need to drill additional holes for screw or anchor fixation, allowing the surgeon to preserve more native bone in the proximal radius. In addition to limiting non-functional retained foreign bodies in the intramedullary canal after revision surgery, the technique also decreases the cost of revision surgery. The additional cost incurred by using another button is described to be as low as $473.33 for the button alone and as high as $4000 when using a new kit.^8^^,^^9^ Finally, there is no need to violate the dorsal cortex, minimizing the risk of injury to the posterior interosseous nerve. There are few risks associated with trying to use the previously placed intramedullary button. It is possible that bony overgrowth or soft tissue interposition in the button could limit successful shuttling of the new suture through the old button (Table I).

**Table I:** Advantages and Disadvantages of the Described Technique

Advantages
No retention of nonfunctioning implants
Cost-effective
Bone preserving and maintained biomechanical strength
No risk of injury to posterior interosseous nerve
Disadvantages
Can be tedious undoing previous knots
Increased cost if suture lasso used
Increased operating room time clearing out drill holes and shuttling suture

Attempting to find and clear out the old drill hole to allow unimpeded suture shuttling may increase time used in the operating room. It is possible that the tissue interposition could preclude any suture shuttling. In this instance, the surgeon must decide whether to leave the old buttons in the intramedullary canal and use new implants, or perform a corticotomy, remove the old buttons, and use alternate methods to repair the distal biceps tendon. Given the morbidity associated with removing intramedullary implants, the more likely solution would be to leave the buttons in the intramedullary canal and use another method of fixation.

When performing this technique, it is crucial to gain excellent exposure of the repair site to take down the knot with minimal compromise to the old repair suture. Once the knot is taken down, it is also crucial to clearly expose the old drill hole and clear out any interposed soft tissue so the suture can effectively shuttle through the old button. We used 2 techniques to shuttle new suture through the old button: a 2-0 Vicryl shuttle stitch and a labral suture passer. The use of a 2-0 shuttle stitch sewn through the old no. 2 suture is more time consuming; however, it is more reliable and cost effective than opening a labral suture passing device. The method of fixation at the index surgery is important to consider when using this technique. If a looped no. 2 suture was used to create a single whipstitch through the tendon, the tails of the old suture will be too short to allow suture shuttling via the suture lasso technique, and the 2-0 Vicryl technique should be used. If the repair was done by first placing the button with suture in it, then running a Krackow-type suture up and down the tendon, then the old no. 2 repair suture tails will likely be long after undoing the whipstitch from the tendon, and the suture lasso technique is easier because tension can be maintained on the old suture as the suture lasso is shuttled through the button (Table 2).

**Table 2:** Pearls and Pitfalls

Pearls
Gentle handling of suture when undoing old knots
Expose and clear out previous drill hole for unimpeded suture shuttling
Test shuttle to ensure smooth sliding before fully shuttling new suture through button
If old suture tails are short after undoing the knot, use the 2-0 Vicryl technique
Pitfalls
Suture fraying and compromise when undoing knots with dental pick
Interposed tissue can inhibit suture shuttling through button
If shuttle suture breaks when trying to pull through and no suture remains in the old button, the technique cannot be performed
New suture will not stay within the suture lasso loop if the tails are too short to keep tension on

## Conclusion

To our knowledge, this is the first report detailing methods to revise a distal biceps repair fixed with intramedullary unicortical buttons and retain the original buttons. This technique is simple and cost effective, minimizes additional morbidity in revision distal biceps surgery, and can potentially be used in revision surgery in other anatomic locations where cortical buttons were used.

## Supplementary Data

ICMJE author disclosure forms

Video 1 Revision distal biceps repair using the original intramedullary unicortical buttons. The patient is supine on a gurney with a hand table extension. The previous incision is used, and the repair site is exposed. Previously tied knots are carefully undone, and the whipstitch in the distal biceps tendon is taken down, with care taken to maintain the integrity of the suture. Once the suture is isolated, the previous drill hole is cleared of interposed soft tissue. A 2-0 Vicryl is then sewn into the old repair suture to act as a shuttling stitch. The 2-0 Vicryl is shuttled through the old button, the old suture is cut off, and then the 2-0 Vicryl is again sewn into a new no. 2 repair suture. The new no. 2 repair suture is shuttled through the button and can then be used to complete the revision distal biceps tendon repair.
