# Supplementary material for: Revision Distal Biceps Tendon Repair Using Original Intramedullary Buttons
Source: Arthrosc Tech. 2023 Jan 18;12(2):e297–300. doi: 10.1016/j.eats.2022.11.006 (PMC9984848; doi:10.1016/j.eats.2022.11.006)
Supplement: ICMJE author disclosure forms [file mmc1.pdf]

Arthroscopy: The Journal of Arthroscopic and Related Surgery

— Instructions —

ICMJE Form for Disclosure of Potential Conflicts of Interest

Each author of the manuscript must separately complete and save this form using his or her name in the file name. Each author's completed form must then be uploaded with the manuscript.

The purpose of this form is to provide readers of your manuscript with information about your other interests that could influence how they receive and understand your work. The form is in four parts:

Section 1. Identifying information

Enter your full name and provide the manuscript title.

Section 2. The work under consideration for publication

This section asks for information about the work that you have submitted for publication. The time frame for this reporting is that of the work itself, from the initial conception and planning to the present. The requested information is about resources that you received, either directly or indirectly (via your institution), to enable you to complete the work. Checking "No" means that you did the work without receiving any financial support from any third party — that is, the work was supported by funds from the same institution that pays your salary and that institution did not receive third-party funds with which to pay you. If you or your institution received funds from a third party to support the work, such as a government granting agency, charitable foundation, or commercial sponsor, check "Yes." Then complete the provide the information requested.

Section 3. Relevant financial activities outside the submitted work

This section asks about your financial relationships with entities in the biomedical arena that could be perceived to influence, or that give the appearance of potentially influencing, what you wrote in the submitted work. You should disclose interactions with ANY entity that could be considered broadly relevant to the work.

Report all sources of revenue paid (or promised to be paid) directly to you or your institution on your behalf over the 36 months prior to submission of the work. This should include all monies from sources with relevance to the submitted work, not just monies from the entity that sponsored the research. Please note that your interactions with the work's sponsor that are outside the submitted work should also be listed here. If there is any question, it is usually better to disclose a relationship than not to do so.

For grants you have received for work outside the submitted work, you should disclose support ONLY from entities that could be perceived to be affected financially by the published work, such as entities or foundations supported by entities that could be perceived to have a financial stake in the outcome. Public funding sources, such as government agencies, charitable foundations, or academic institutions, need not be disclosed. For example, if a government agency sponsored a study in which you have been involved and drugs were provided by a pharmaceutical company, you need only list the pharmaceutical company.

Section 4. Other relationships

Use this section to report other relationships or activities that readers could perceive to have influenced, or that give the appearance of potentially influencing, what you wrote in the submitted work.

ICMJE Form for Disclosure of Potential Conflicts of Interest

Section 1. Identifying Information

1. Given Name ____________Bryan ____ 2. Surname _____Adams_______________________
3. Are you the corresponding author? Yes __X_ No___
4. Effective Date ___08 AUG 2022________________________
5. Manuscript Title ___Revision distal biceps tendon repair utilizing original intramedullary buttons___________

Section 2. The Work Under Consideration for Publication

Did you or your institution at any time receive payment or services from a third party for any aspect of the submitted work (including but not limited to grants, data monitoring board, study design, manuscript preparation, statistical analysis, etc…)?

Complete each item by typing an X in answer yes or not and completing the information requested if an answer is Yes. If you have more than one relationship, add lines.

1. Grant

_**x**__No ___Yes, money paid to you ___Yes, money paid to institution* Name of entity___
Comments†___

2. Consulting fee or honorarium

_**x**__No ___Yes, money paid to you ___Yes, money paid to institution* Name of entity___
Comments†___

3. Support for travel to meetings for the study or other purposes

_ **x** __No ___Yes, money paid to you ___Yes, money paid to institution* Name of entity___
Comments†___

4. Fees for participation in review activities such as data monitoring boards, statistical analysis, end-point committees, and the like

_ **x** __No ___Yes, money paid to you ___Yes, money paid to institution* Name of entity___
Comments†___

5. Payment for writing or reviewing the manuscript

_ **x** __No ___Yes, money paid to you ___Yes, money paid to institution* Name of entity___
Comments†___

6. Provision of writing assistance, medicines, equipment, or administrative support

__ **x** _No ___Yes, money paid to you ___Yes, money paid to institution* Name of entity___
Comments†___

7. Other

_ **x** __No ___Yes, money paid to you ___Yes, money paid to institution* Name of entity___
Comments†___

* This means money that your institution received for your efforts on this study.
† Use this section to provide any needed explanation.

Section 3. Relevant financial activities outside the submitted work

1. Board membership

_ **x** __No ___Yes, money paid to you ___Yes, money paid to institution* Name of entity___
Comments___

2. Consultancy

_ **x** __No ___Yes, money paid to you ___Yes, money paid to institution* Name of entity___
Comments___

3. Employment

_ **x** __No ___Yes, money paid to you ___Yes, money paid to institution* Name of entity___
Comments___

4. Expert testimony

_ **x** __No ___Yes, money paid to you ___Yes, money paid to institution* Name of entity___
Comments___

5. Grants/grants pending

_ **x** __No ___Yes, money paid to you ___Yes, money paid to institution* Name of entity___
Comments___

6. Payment for lectures including service on speakers bureaus

_ **x** __No ___Yes, money paid to you ___Yes, money paid to institution* Name of entity___
Comments___

7. Payment for manuscript preparation

_ **x** __No ___Yes, money paid to you ___Yes, money paid to institution* Name of entity___
Comments___

8. Patents (planned, pending or issued)

_ **x** __No ___Yes, money paid to you ___Yes, money paid to institution* Name of entity___
Comments___

9. Royalties

__ **x** _No ___Yes, money paid to you ___Yes, money paid to institution* Name of entity___
Comments___

10. Payment for development of educational presentations

_ **x** __No ___Yes, money paid to you ___Yes, money paid to institution* Name of entity___
Comments___

11. Stock/stock options

_ **x** __No ___Yes, money paid to you ___Yes, money paid to institution* Name of entity___
Comments___

12. Travel/accommodations/ meeting expenses unrelated to activities listed**

_ **x** __No ___Yes, money paid to you ___Yes, money paid to institution* Name of entity___
Comments___

13. Other (err on the side of full disclosure)

__ **x** _No ___Yes, money paid to you ___Yes, money paid to institution* Name of entity___
Comments___

** For example, if you report a consultancy above there is no need to report travel related to that consultancy on this line.

Section 4. Other relationships

Are there other relationships or activities that readers could perceive to have influenced, or that give the appearance of potentially influencing, what you wrote in the submitted work?

_ **x** __No other relationships/conditions/circumstances that present a potential conflict of interest

___Yes, the following relationships/conditions/circumstances are present (explain below):

>>>>>>>>>>><<<<<<<<<<<

The International Committee of Medical Journal Editors

The ICMJE Disclosure of Potential Conflicts of Interest Form was adopted by *Arthroscopy: The Journal of Arthroscopic and Related Surgery* along with 17 other leading orthopaedic journals at the 2011 annual meeting of the American Academy of Orthopaedic Surgeons.

ICMJE Form for Disclosure of Potential Conflicts of Interest

Section 1. Identifying Information

1. Given Name ____________Bobby ____ 2. Surname _____Yow_______________________
3. Are you the corresponding author? Yes ___ No_x__
4. Effective Date ___08 AUG 2022________________________
5. Manuscript Title ___Revision distal biceps tendon repair utilizing original intramedullary buttons___________

Section 2. The Work Under Consideration for Publication

Did you or your institution at any time receive payment or services from a third party for any aspect of the submitted work (including but not limited to grants, data monitoring board, study design, manuscript preparation, statistical analysis, etc…)?

Complete each item by typing an X in answer yes or not and completing the information requested if an answer is Yes. If you have more than one relationship, add lines.

1. Grant

_**x**__No ___Yes, money paid to you ___Yes, money paid to institution* Name of entity___
Comments†___

2. Consulting fee or honorarium

_**x**__No ___Yes, money paid to you ___Yes, money paid to institution* Name of entity___
Comments†___

3. Support for travel to meetings for the study or other purposes

_ **x** __No ___Yes, money paid to you ___Yes, money paid to institution* Name of entity___
Comments†___

4. Fees for participation in review activities such as data monitoring boards, statistical analysis, end-point committees, and the like

_ **x** __No ___Yes, money paid to you ___Yes, money paid to institution* Name of entity___
Comments†___

5. Payment for writing or reviewing the manuscript

_ **x** __No ___Yes, money paid to you ___Yes, money paid to institution* Name of entity___
Comments†___

6. Provision of writing assistance, medicines, equipment, or administrative support

__ **x** _No ___Yes, money paid to you ___Yes, money paid to institution* Name of entity___
Comments†___

7. Other

_ **x** __No ___Yes, money paid to you ___Yes, money paid to institution* Name of entity___
Comments†___

* This means money that your institution received for your efforts on this study.
† Use this section to provide any needed explanation.

Section 3. Relevant financial activities outside the submitted work

1. Board membership

_ **x** __No ___Yes, money paid to you ___Yes, money paid to institution* Name of entity___ Comments___

2. Consultancy

_ **x** __No ___Yes, money paid to you ___Yes, money paid to institution* Name of entity___ Comments___

3. Employment

_ **x** __No ___Yes, money paid to you ___Yes, money paid to institution* Name of entity___ Comments___

4. Expert testimony

_ **x** __No ___Yes, money paid to you ___Yes, money paid to institution* Name of entity___ Comments___

5. Grants/grants pending

_ **x** __No ___Yes, money paid to you ___Yes, money paid to institution* Name of entity___ Comments___

6. Payment for lectures including service on speakers bureaus

_ **x** __No ___Yes, money paid to you ___Yes, money paid to institution* Name of entity___ Comments___

7. Payment for manuscript preparation

_ **x** __No ___Yes, money paid to you ___Yes, money paid to institution* Name of entity___ Comments___

8. Patents (planned, pending or issued)

_ **x** __No ___Yes, money paid to you ___Yes, money paid to institution* Name of entity___ Comments___

9. Royalties

__ **x** _No ___Yes, money paid to you ___Yes, money paid to institution* Name of entity___ Comments___

10. Payment for development of educational presentations

_ **x** __No ___Yes, money paid to you ___Yes, money paid to institution* Name of entity___ Comments___

11. Stock/stock options

_ **x** __No ___Yes, money paid to you ___Yes, money paid to institution* Name of entity___
Comments___

12. Travel/accommodations/ meeting expenses unrelated to activities listed**

_ **x** __No ___Yes, money paid to you ___Yes, money paid to institution* Name of entity___
Comments___

13. Other (err on the side of full disclosure)

__ **x** _No ___Yes, money paid to you ___Yes, money paid to institution* Name of entity___
Comments___

** For example, if you report a consultancy above there is no need to report travel related to that consultancy on this line.

Section 4. Other relationships

Are there other relationships or activities that readers could perceive to have influenced, or that give the appearance of potentially influencing, what you wrote in the submitted work?

_ **x** __No other relationships/conditions/circumstances that present a potential conflict of interest

___Yes, the following relationships/conditions/circumstances are present (explain below):

>>>>>>>>>>><<<<<<<<<<<

The International Committee of Medical Journal Editors

The ICMJE Disclosure of Potential Conflicts of Interest Form was adopted by *Arthroscopy: The Journal of Arthroscopic and Related Surgery* along with 17 other leading orthopaedic journals at the 2011 annual meeting of the American Academy of Orthopaedic Surgeons.

ICMJE Form for Disclosure of Potential Conflicts of Interest

Section 1. Identifying Information

1. Given Name ____________Daniels ____ 2. Surname _____Christopher_______________________
3. Are you the corresponding author? Yes ___ No_xx__
4. Effective Date ___08 AUG 2022________________________
5. Manuscript Title: Revision distal biceps tendon repair utilizing original intramedullary buttons

Section 2. The Work Under Consideration for Publication

Did you or your institution at any time receive payment or services from a third party for any aspect of the submitted work (including but not limited to grants, data monitoring board, study design, manuscript preparation, statistical analysis, etc…)?

Complete each item by typing an X in answer yes or not and completing the information requested if an answer is Yes. If you have more than one relationship, add lines.

1. Grant

_**x**__No ___Yes, money paid to you ___Yes, money paid to institution* Name of entity___
Comments†___

2. Consulting fee or honorarium

_**x**__No ___Yes, money paid to you ___Yes, money paid to institution* Name of entity___
Comments†___

3. Support for travel to meetings for the study or other purposes

_ **x** __No ___Yes, money paid to you ___Yes, money paid to institution* Name of entity___
Comments†___

4. Fees for participation in review activities such as data monitoring boards, statistical analysis, end-point committees, and the like

_ **x** __No ___Yes, money paid to you ___Yes, money paid to institution* Name of entity___
Comments†___

5. Payment for writing or reviewing the manuscript

_ **x** __No ___Yes, money paid to you ___Yes, money paid to institution* Name of entity___
Comments†___

6. Provision of writing assistance, medicines, equipment, or administrative support

__ **x** _No ___Yes, money paid to you ___Yes, money paid to institution* Name of entity___
Comments†___

7. Other

_ **x** __No ___Yes, money paid to you ___Yes, money paid to institution* Name of entity___
Comments†___

* This means money that your institution received for your efforts on this study.
† Use this section to provide any needed explanation.

Section 3. Relevant financial activities outside the submitted work

1. Board membership

_ **x** __No ___Yes, money paid to you ___Yes, money paid to institution* Name of entity___ Comments___

2. Consultancy

_ **x** __No ___Yes, money paid to you ___Yes, money paid to institution* Name of entity___ Comments___

3. Employment

_ **x** __No ___Yes, money paid to you ___Yes, money paid to institution* Name of entity___ Comments___

4. Expert testimony

_ **x** __No ___Yes, money paid to you ___Yes, money paid to institution* Name of entity___ Comments___

5. Grants/grants pending

_ **x** __No ___Yes, money paid to you ___Yes, money paid to institution* Name of entity___ Comments___

6. Payment for lectures including service on speakers bureaus

_ **x** __No ___Yes, money paid to you ___Yes, money paid to institution* Name of entity___ Comments___

7. Payment for manuscript preparation

_ **x** __No ___Yes, money paid to you ___Yes, money paid to institution* Name of entity___ Comments___

8. Patents (planned, pending or issued)

_ **x** __No ___Yes, money paid to you ___Yes, money paid to institution* Name of entity___ Comments___

9. Royalties

__ **x** _No ___Yes, money paid to you ___Yes, money paid to institution* Name of entity___ Comments___

10. Payment for development of educational presentations

_ **x** __No ___Yes, money paid to you ___Yes, money paid to institution* Name of entity___ Comments___

11. Stock/stock options

_ **x** __No ___Yes, money paid to you ___Yes, money paid to institution* Name of entity___ Comments___

12. Travel/accommodations/ meeting expenses unrelated to activities listed**

_ **x** __No ___Yes, money paid to you ___Yes, money paid to institution* Name of entity___
Comments___

13. Other (err on the side of full disclosure)

__ **x** _No ___Yes, money paid to you ___Yes, money paid to institution* Name of entity___
Comments___

** For example, if you report a consultancy above there is no need to report travel related to that consultancy on this line.

Section 4. Other relationships

Are there other relationships or activities that readers could perceive to have influenced, or that give the appearance of potentially influencing, what you wrote in the submitted work?

_ **x** __No other relationships/conditions/circumstances that present a potential conflict of interest

___Yes, the following relationships/conditions/circumstances are present (explain below):

>>>>>>>>>>><<<<<<<<<<<

The International Committee of Medical Journal Editors

The ICMJE Disclosure of Potential Conflicts of Interest Form was adopted by *Arthroscopy: The Journal of Arthroscopic and Related Surgery* along with 17 other leading orthopaedic journals at the 2011 annual meeting of the American Academy of Orthopaedic Surgeons.

ICMJE Form for Disclosure of Potential Conflicts of Interest

Section 1. Identifying Information

1. Given Name ____________Morgan ____ 2. Surname _____Emily_______________________
3. Are you the corresponding author? Yes ___ No_x__
4. Effective Date ___08 AUG 2022________________________
5. Manuscript Title: Revision distal biceps tendon repair utilizing original intramedullary buttons

Section 2. The Work Under Consideration for Publication

Did you or your institution at any time receive payment or services from a third party for any aspect of the submitted work (including but not limited to grants, data monitoring board, study design, manuscript preparation, statistical analysis, etc…)?

Complete each item by typing an X in answer yes or not and completing the information requested if an answer is Yes. If you have more than one relationship, add lines.

1. Grant

_**x**__No ___Yes, money paid to you ___Yes, money paid to institution* Name of entity___
Comments†___

2. Consulting fee or honorarium

_**x**__No ___Yes, money paid to you ___Yes, money paid to institution* Name of entity___
Comments†___

3. Support for travel to meetings for the study or other purposes

_ **x** __No ___Yes, money paid to you ___Yes, money paid to institution* Name of entity___
Comments†___

4. Fees for participation in review activities such as data monitoring boards, statistical analysis, end-point committees, and the like

_ **x** __No ___Yes, money paid to you ___Yes, money paid to institution* Name of entity___
Comments†___

5. Payment for writing or reviewing the manuscript

_ **x** __No ___Yes, money paid to you ___Yes, money paid to institution* Name of entity___
Comments†___

6. Provision of writing assistance, medicines, equipment, or administrative support

__ **x** _No ___Yes, money paid to you ___Yes, money paid to institution* Name of entity___
Comments†___

7. Other

_ **x** __No ___Yes, money paid to you ___Yes, money paid to institution* Name of entity___
Comments†___

* This means money that your institution received for your efforts on this study.
† Use this section to provide any needed explanation.

Section 3. Relevant financial activities outside the submitted work

1. Board membership

_ **x** __No ___Yes, money paid to you ___Yes, money paid to institution* Name of entity___
Comments___

2. Consultancy

_ **x** __No ___Yes, money paid to you ___Yes, money paid to institution* Name of entity___
Comments___

3. Employment

_ **x** __No ___Yes, money paid to you ___Yes, money paid to institution* Name of entity___
Comments___

4. Expert testimony

_ **x** __No ___Yes, money paid to you ___Yes, money paid to institution* Name of entity___
Comments___

5. Grants/grants pending

_ **x** __No ___Yes, money paid to you ___Yes, money paid to institution* Name of entity___
Comments___

6. Payment for lectures including service on speakers bureaus

_ **x** __No ___Yes, money paid to you ___Yes, money paid to institution* Name of entity___
Comments___

7. Payment for manuscript preparation

_ **x** __No ___Yes, money paid to you ___Yes, money paid to institution* Name of entity___
Comments___

8. Patents (planned, pending or issued)

_ **x** __No ___Yes, money paid to you ___Yes, money paid to institution* Name of entity___
Comments___

9. Royalties

__ **x** _No ___Yes, money paid to you ___Yes, money paid to institution* Name of entity___
Comments___

10. Payment for development of educational presentations

_ **x** __No ___Yes, money paid to you ___Yes, money paid to institution* Name of entity___
Comments___

11. Stock/stock options

_ **x** __No ___Yes, money paid to you ___Yes, money paid to institution* Name of entity___
Comments___

12. Travel/accommodations/ meeting expenses unrelated to activities listed**

_ **x** __No ___Yes, money paid to you ___Yes, money paid to institution* Name of entity___ Comments___

13. Other (err on the side of full disclosure)

__ **x** _No ___Yes, money paid to you ___Yes, money paid to institution* Name of entity___ Comments___

** For example, if you report a consultancy above there is no need to report travel related to that consultancy on this line.

Section 4. Other relationships

Are there other relationships or activities that readers could perceive to have influenced, or that give the appearance of potentially influencing, what you wrote in the submitted work?

_ **x** __No other relationships/conditions/circumstances that present a potential conflict of interest

___Yes, the following relationships/conditions/circumstances are present (explain below):

>>>>>>>>>>><<<<<<<<<<<

The International Committee of Medical Journal Editors

The ICMJE Disclosure of Potential Conflicts of Interest Form was adopted by *Arthroscopy: The Journal of Arthroscopic and Related Surgery* along with 17 other leading orthopaedic journals at the 2011 annual meeting of the American Academy of Orthopaedic Surgeons.
